# Proteomics analysis of a human brain sample from a mucolipidosis type IV patient reveals pathophysiological pathways

**DOI:** 10.1186/s13023-021-01679-7

**Published:** 2021-01-21

**Authors:** Ayelet Vardi, Amir Pri-Or, Noa Wigoda, Yulia Grishchuk, Anthony H. Futerman

**Affiliations:** 1 Department of Biomolecular Sciences, Weizmann Institute of Science, 76100 Rehovot, Israel; 2 The Nancy and Stephen Grand Israel National Center for Personalized Medicine, Weizmann Institute of Science, 76100 Rehovot, Israel; 3 The Life Sciences Core Facilities, Weizmann Institute of Science, 76100 Rehovot, Israel; 4 Center for Genomic Medicine and Department of Neurology, Massachusetts General Hospital Research Institute, Harvard Medical School, 185 Cambridge St., Boston, MA 02114 USA

**Keywords:** Lysosome, TRPML1, Brain, Neuropathology, Autophagy, Neuroinflammation, Calbindin

## Abstract

**Background:**

Mucolipidosis type IV (MLIV), an ultra-rare neurodevelopmental and neurodegenerative disorder, is caused by mutations in the *MCOLN1* gene, which encodes the late endosomal/lysosomal transient receptor potential channel TRPML1 (mucolipin 1). The precise pathophysiogical pathways that cause neurological disease in MLIV are poorly understood. Recently, the first post-mortem brain sample became available from a single MLIV patient, and in the current study we performed mass spectrometry (MS)-based proteomics on this tissue with a view to delineating pathological pathways, and to compare with previously-published data on MLIV, including studies using the *Mcoln1*^*−/−*^ mouse.

**Results:**

A number of pathways were altered in two brain regions from the MLIV patient, including those related to the lysosome, lipid metabolism, myelination, cellular trafficking and autophagy, mTOR and calmodulin, the complement system and interferon signaling. Of these, levels of some proteins not known previously to be associated with MLIV were altered, including APOD, PLIN4, ATG and proteins related to interferon signaling. Moreover, when proteins detected by proteomics in the human brain were compared with their orthologs detected in the *Mcoln1*^*−/−*^ mouse by RNAseq, the results were remarkably similar. Finally, analysis of proteins in human and mouse CSF suggest that calbindin 1 and calbindin 2 might be useful as biomarkers to help chart the course of disease development.

**Conclusions:**

Despite the sample size limitations, our findings are consistent with the relatively general changes in lysosomal function previously reported in MLIV, and shed light on new pathways of disease pathophysiology, which is required in order to understand the course of disease development and to determine the efficacy of therapies when they become available for this devastating disease.

## Introduction

Mucolipidosis type IV (MLIV), an ultra-rare lysosomal storage disease (LSD) primarily found in the Ashkenazi Jewish population [1], is caused by mutations in the *MCOLN1* gene, which encodes the late endosomal/lysosomal transient receptor potential channel TRPML1 (mucolipin 1) [2]. Over two dozen mutations in *MCOLN1* have been identified which lead to MLIV [3]. TRPML1 is a non-selective cation channel that regulates lysosomal ion content [4, 5]. TRPML1 also plays a role in autophagy regulation, intracellular trafficking and in the mechanistic target of rapamycin (mTOR)/transcription factor EB (TFEB) signaling axis [6, 7]. MLIV is a neurodevelopmental and neurodegenerative disorder with severe psychomotor developmental delay, impaired gastric function, mental retardation, motor deficits and progressive visual impairment [1, 8]. At present there is no therapy for MLIV and our understanding of brain pathophysiology is limited due to the lack of human tissue samples available for research.

We recently obtained brain tissue and cerebrospinal fluid (CSF) from a 36-year-old male who died from MLIV-related complications. This is the first time that post-mortem brain tissue has been made available from a MLIV patient. We performed mass spectrometry (MS)-based proteomics on this tissue with a view to delineating pathological pathways. Moreover, we compared our data with that obtained using a mouse model of MLIV, namely the *Mcoln1*^*−/−*^ mouse [9, 10], which further validated that the *Mcoln1*^*−/−*^ mouse is a genuine animal model of MLIV, and of more importance, allowed us to identify some novel pathological components and pathways in the human MLIV brain tissue, such as activation of complement and interferon (IFN) pathways and lipid droplet and lipoprotein metabolism. Along with the identification of a potential biomarker (calbindin 2) for early disease development in the CSF, our study paves the way for further understanding of the molecular pathways that lead to disease pathophysiology in MLIV, a pre-requisite for development of putative therapies.

## Methods

### Human brain and CSF samples

Human brain and CSF samples were obtained from the NIH NeuroBioBank Brain and Tissue Repository at the University of Maryland, Baltimore. The post mortem MLIV patient was a 36 year-old male and the control was a 34-year-old healthy male who died from unrelated causes.

### Brain tissue MS-based proteomics

Brain tissue was lysed using a GentleMACS dissociator (Miltenyi Biotec, Bergisch Gladbach, Germany) in 50 mM Tris HCl containing 5% sodium dodecyl sulfate (SDS) and supplemented with a protease inhibitor cocktail (1:200, Sigma-Aldrich, Missouri, USA). Homogenates were incubated with 5 mM dithiothreitol for 1 h at 56 °C followed by 10 mM iodoacetamide in the dark at room temperature for 45 min. Samples were loaded onto S-Trap microcolumns (Protifi, New-York, USA) and subjected to tryptic digestion [11, 12]. Samples were vacuum dried and stored at -80 °C. The resulting peptides were analyzed by nanoflow liquid chromatography (NanoAcquity, Waters, Milford, MA, USA) coupled to high resolution, high mass accuracy mass spectrometry (Fusion Lumos, Thermo Scientific, Waltham, MA, USA) and sequentially analyzed in discovery mode. Raw data was processed with MaxQuant v1.6.0.16 [13]. Data was analyzed with the Andromeda search engine against the human proteome database (www.uniprot.com) and appended with common laboratory protein contaminants. Quantitative comparisons were calculated using Perseus v1.6.0.7. Pathway analysis was performed using Gene Analytics [14]. Proteins were categorized based on the Human Protein Atlas [15] (http://www.proteinatlas.org).

### CSF MS-based proteomics

Samples were lysed with 8 M urea and incubated at room temperature for 30 min prior to incubation with 5 mM dithiothreitol (Sigma-Aldrich) at room temperature for 1 h, followed by alkylation with 10 mM iodoacetamide (Sigma-Aldrich) in the dark at room temperature for 45 min. Samples were diluted in 2 M urea with 50 mM ammonium bicarbonate. Proteins were digested with trypsin (50:1; w/vol) (Promega, Madison, WI, USA) overnight at 37 °C followed by a second trypsin digestion for 4 h. Digestion was terminated using trifluoracetic acid (1% final concentration). Peptides were desalted using an Oasis hydrophilic lipophilic balance column (Waters) and then vacuum dried and stored at -80 °C. The resulting peptides were analyzed using nanoflow liquid chromatography (nanoACQUITY, Waters) coupled to high resolution, high mass accuracy mass spectrometry (Q Exactive HFX for mouse CSF or Fusion Lumos for human CSF, Thermo Scientific). Samples were analyzed in random order in discovery mode. The raw data was processed with MaxQuant v1.6.0.16 [13] and analyzed with the Andromeda search engine against the mouse or human proteome database (www.uniprot.com), and appended with common laboratory protein contaminants. Quantitative comparisons were calculated using Perseus v1.6.0.7. Pathway analysis was done using Gene Analytics [14].

### Mice

*Mcoln1*^*−/−*^ mice on a C57Bl/6 J background [10] were used. Littermates (*Mcoln1*^+*/*+^ or *Mcoln1*^±^) were used as controls and both females and males were used in all studies. Genotyping was performed by PCR using genomic DNA extracted from mouse tails. Mice were maintained in the Experimental Animal Center of the Weizmann Institute of Science. Animal experiments were approved by the Weizmann Institute Institutional Animal Care and Use Committee.

### Mouse CSF

1-, 2-, 3- and 7-month-old *Mcoln*^±^ or *Mcoln1*^*−/−*^ mice were anesthetized using 200 mg/ml sodium pentobarbital and CSF aspirated from the cisterna magna using a glass needle. CSF samples were kept at -80 °C.

### RNAseq

Brain tissue was homogenized using a GentleMACS dissociator and mRNA was isolated using the RNeasy mini kit (Qiagen GmbH, Hilden, Germany). RNA concentration (ratio 260/230 and 260/280 nm) was measured using a NanoDrop ND-1000 (Thermo Scientific, Waltham, MA, USA) and RNA integrity evaluated using an RNA screen tape on a Tapestation 2200 (Agilent, Santa Clara, California, USA). A bulk variation of MARSseq [16] was used to construct RNAseq libraries. Sequencing was performed using an Illumina Nextseq-500 75 cycle high output kit (Illumina, San Diego, California, USA; paired end sequencing). Raw reads were mapped to the *Mus musculus* genome (mm10) using STAR. Only reads which mapped uniquely to genes were considered for further analysis. Differentially-expressed genes (DEGs) were selected using a 1.5-fold change cutoff between two populations and adjusted *p* value for multiple gene testing of < 0.05 [17]. Pathway analysis was performed using Gene Analytics [14]. Ortholog conversion was done using HUGO Gene Nomenclature Committee.

### Enzyme-linked immunosorbent assay

Calbindin 2 was quantified using a Calertinin ELISA kit (Abcam, Cambridge, MA) according to manufacturer’s instructions. Samples were analyzed in triplicate.

## Results

MS-based proteomics identified 3968 proteins in the cerebellum (CB) (with more than one unique peptide) and 3936 in the cerebral cortex (CRB). Of these, 959 were increased and 1512 decreased in MLIV compared to the control in the CB, and 824 proteins increased and 1168 decreased in the CRB (ratio > 1.5). 2113 proteins were detected in the CSF, with 1018 elevated (ratio > 1.5) and 526 proteins decreased in MLIV compared to the control. Due to the minimal sample size, label-free, relative quantification was calculated by dividing protein intensity in the MLIV sample by protein intensity in the control [18, 19]. When a ratio could not be calculated, i.e., protein levels were below the level of detection in one or other of the samples, changes are indicated by ‘ + ’ for a protein that was detected only in the MLIV sample, and by ‘-’ when the protein was detected only in the control sample (see Tables 1, 2, 3, 4, 5, 6 and 7 and 9).

**Table 1:** Lysosomal proteins in human brain and CSF

Protein	CB	CRB	CSF
*Ratio (MLIV/Con)*			
*Lysosomal hydrolases*			
HEXA	1.77	2.93	0.90
HEXB	5.96	3.95	6.96
NPC1	−	3.93	n.d
NPC2	2.58	4.34	2.59
GNS	2.94	3.01	+
GM2A	3.46	9.24	2.66
GLB1	3.98	2.05	n.d
GAA	4.56	2.74	4.90
GUSB	7.07	+	n.d
ASAH1	6.41	5.11	2.61
PLBD2	0.45	2.51	n.d
TPP1	2.15	2.18	0.70
ARSA	3.56	2.29	1.70
LIPA	1.39	4.49	n.d
GALNS	−	−	n.d
*Cathepsins*			
CTSA	+	1.49	+
CTSB	2.07	1.67	1.23
CTSD	1.21	1.46	1.30
CTSH	3.08	1.84	2.11
CTSL	0.40	2.73	1.09
CTSZ	0.81	2.27	3.17
*Lysosomal proteins*			
LAMP1	2.82	2.44	
LAMP2	3.31	4.71	1.53
PRCP	7.52	11.7	2.71
PSAP	3.37	3.92	7.30
CAV1	2.99	1.09	0.07
ACP2	0.88	3.19	n.d
SCARB2	2.82	2.13	n.d
DNASE2	1.09	+	n.d
NHLRC3	+	+	n.d
PPT1	1.88	2.58	1.39
FTH1	1.27	1.86	4.65
FTL	0.92	1.96	2.80
LMBRD1	−	n.d	n.d

A total of 42 lysosomal proteins [73] were detected. The proteins listed in the Table are those that differ between the control and MLIV in one or other tissue. When a ratio could not be calculated (i.e., a value of zero was obtained in either the control or in MLIV), changes are indicated by + for a protein that was detected only in the MLIV sample, and by—when the protein was detected only in the control sample; n.d., not detected in either sample. Con, control; CB, cerebellum; CRB, cerebrum

**Fig.1:**
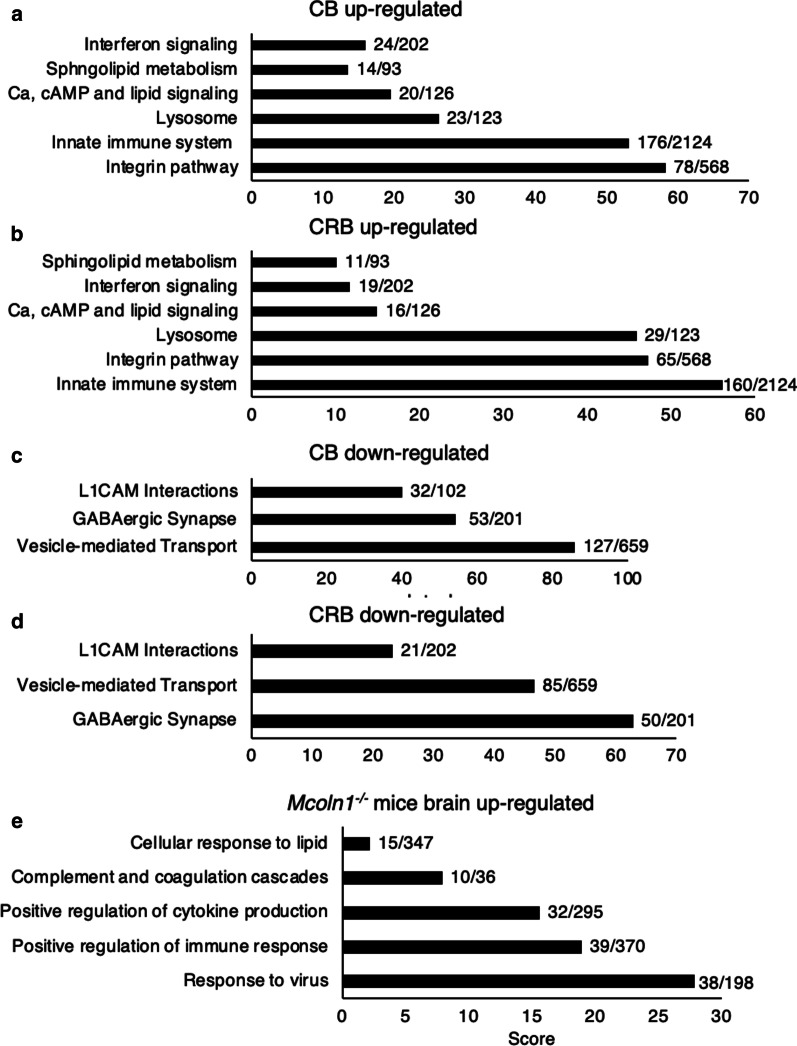
Up- and down-regulated pathways in post mortem MLIV brain and in 7-month-old *Mcoln1*^*−/−*^ mouse brain. Pathway analysis of up-(**a**, **b**)-and down-regulated **c**, **d** proteins (ratio of MLIV/ control > 1.5) in the cerebellum (CB) and cerebrum (CRB). **e** Pathway analysis of DEGs (fold-change > 1.5, *p* adjusted < 0.05) in 7 month-old *Mcoln1*^*−/−*^ mice (n = 5) versus *Mcoln1*^±^ mice (n = 5). The number of identified proteins out of total proteins associated with each pathway are shown

To obtain an overview of changes in the MLIV brain, we first interrogated the proteomics data by pathway analysis. Identical pathways (i.e. sphingolipid metabolism, interferon signaling, the lysosome and others, see Fig. 1a, b) were upþregulated in both the CB and the CRB and likewise similar pathways were down-regulated in the CB and CRB (Fig. 1c, d). The latter includes the GABAergic pathway (including GABRA1, GABRA3, GABRB1, GABRB2, GABBR2) and other neuronal genes (GRIN2B, HPCA, NEGR1, GPM6A, and NPTX1; not shown), along with proteins associated with L1 cell adhesion (L1CAM, Fig. 1.c, d), which plays a role in nervous system development [20]. The identification of similar pathways in two distinct brain regions adds validity to the accuracy of the data, which is taken from one MLIV brain and one control brain, but analyzed experimentally in two different brain areas from each sample. Moreover, the up- and down-regulated pathways are largely consistent with what is known to date on MLIV pathophysiology in *Mcoln1*^*−/−*^ mice [3, 7].

We next examined individual proteins associated with some of these pathways. Levels of most of the lysosomal proteins that were detected by MS increased in MLIV brain including lysosomal hydrolases, cathepsins and a number of other lysosomal proteins (Table 1). Less lysosomal proteins were detected in the CSF than in the CB and CRB, but levels of all detected proteins were similarly increased. The up-regulation of lysosomal proteins is consistent with the broad lysosomal defects resulting from the loss of TRPML1 function that underlies MLIV pathology [21].

Levels of a number of proteins in the sphingolipid metabolic pathway increased in MLIV CB and CRB (although most were not detected in the CSF), as were some proteins associated with cholesterol metabolism (MVD, MVK, MSMO1, SEC14L2), which were elevated in the CB but not CRB (Table 2), also consistent with some of the changes in lipid metabolism previously reported in MLIV [22, 23]. Levels of PLIN4, a protein associated with lipoprotein metabolism and lipid droplets increased, as did APOD (Table 2).

**Table 2:** Proteins related to lipid metabolism in human brain and CSF

Protein	CB	CRB	CSF
*Ratio (MLIV/Con)*			
S1PR1	n.d	+	n.d
S1PR5	n.d	+	n.d
CERS1	1.71	2.01	n.d
KDSR	2.93	4.35	n.d
GALC	+	2.97	n.d
SPHK2	+	1.03	n.d
MVD	2.84	1.31	0.68
MVK	2.08	0.93	n.d
MSMO1	+	0.91	n.d
SEC14L2	3.54	1.51	3.18
SGPP1	n.d	−	n.d
SMPD3	−	0.71	n.d
GBA2	−	0.61	n.d
PLIN3	0.86	2.72	1.46
PLIN4	+	7.41	n.d
APOA1	0.09	0.44	0.15
APOA2	−	1.31	0.20
APOA4	0.53	0.87	0.45
APOB	0.62	n.d	0.20
APOC3	−	−	−
APOD	5.21	10.7	0.26
APOE	0.36	1.12	1.27
APOH	0.81	+	0.06

A total of 18 proteins related to SL metabolism, 6 related to cholesterol, 4 related to ganglioside metabolism, 3 related to lipid droplet and 12 related to apolipoprotein metabolism were detected. The proteins listed in the Table are those that differ between the control and MLIV in one or other tissue. See Table 1 for explanation of the symbols

Changes in lipid metabolism are often related to disrupted myelination [24] which is also detected in MLIV [25–27]. Interestingly, levels of proteins associated with myelination were reduced in the CB, but not in the CRB in the MLIV brain, where their number was increased (Table 3). The reason for the latter is not entirely clear. In the *Mcoln1*^*−/−*^ mouse, myelination defects are found primarily in the CRB [9, 28]; however, our data suggest a regional variability in hypomyelination in different human brain regions, and reduced levels of myelin proteins may reflect degeneration in the cerebellum in this patient as has been reported for other older MLIV patients [26].

**Table 3:** Myelination proteins in human MLIV tissues

Protein	CB	CRB	CSF
*Ratio (MLIV/Con)*			
MOG	0.44	2.84	n.d
MAG	0.37	2.44	n.d
MOBP^1^	0.02	3.35	n.d
LGI4	0.46	0.83	n.d
CNTNAP1	0.46	1.15	n.d
PLP1	1.14	3.49	0.44
PLLP	−	2.41	n.d
ARHGEF10L	1.17	−	n.d
CNP	0.49	2.91	0.96

12 proteins were detected. The proteins listed in the Table are those that differ between the control and MLIV. See Table 1 for explanation of the symbols

^1^ Only one peptide was identified for this protein

TRPML1 has been suggested to indirectly regulate membrane traffic [23, 29]. A relatively large number of proteins associated with trafficking between the ER, Golgi and the lysosome decreased, mainly in the CB, with many of the proteins detected only in the control, but not in the MLIV sample (Table 4). Likewise, changes were detected in a number of autophagy-related proteins (Table 4), consistent with the constitutive block of autophagy reported in MLIV [30]. Several proteins related to autophagy were elevated in MLIV including the autophagosome markers MAP1LC3A and MAP1LC3B [31], indicative of the inhibited autophagosome degradation of lysosomes, whereas a number of ATG proteins, which regulate autophagosome formation [32] were down-regulated (Table 4), supporting the notion that lysosomes are unable to fuse with autophagosomes in MLIV [30]. Among the key regulators of autophagy is TFEB [33]. The activity and localization of TFEB is regulated by mechanistic target of rapamycin (mTOR) phosphorylation. TRPML1 has been suggested to be part of a feedback loop between mTOR and TFEB [34, 35] which could explain the elevation of LAMTOR proteins in MLIV CB and in MLIV CRB (Table 5). Moreover, lysosomal calcium release through TRPML1 is required for mTORC1 activation [36] and TRPML1 is essential for MTORC1 recruitment and activation in a Ca2 + -calmodulin-dependent manner [37], consistent with the decrease in levels of several calmodulin-associated proteins in MLIV (Table 5).

**Table 4:** Proteins associated with cellular trafficking and autophagy in human MLIV brain

Protein	CB	CRB
Ratio (MLIV/Con)		
*Cellular trafficking*		
TMEM106B	1.27	3.2
GGA3	−	0.57
GORS2	−	0.91
TRAPPC2L	−	−
TRAPPC11	−	1.03
TRAPPC6B	−	1.06
TRAPPC9	−	1.11
SYT11	−	−
SNX9	−	1.48
SNAP29	−	0.78
RAB9A	−	+
RAB9B	0.09	−
VAMP7	n.d	−
VPS41	0.79	2.11
VTI1B	0.63	0.65
WDR81	−	+
*Autophagy*		
ACBD5	+	1.33
ATG3	4.81	2.04
DCN	2.31	+
MAP1LC3A	2.23	3.09
MAP1LC3B/2^1^	+	+
SQSTM1	1.15	2.55
ATG4B	−	n.d
ATG5	−	0.9
ATG7	−	1.51
ATG16L1	−	−
ATG2B	−	0.68
ATG13	−	−
WIPI2	−	0.71
SMCR8	−	−

23 proteins were detected related to cellular trafficking and 31 proteins related to autophagy (27 had > 1 unique peptide). The proteins listed in the Table are those that differ between the control and MLIV. See Table 1 for explanation of the symbols

^1^ The detected peptides cannot distinguish between MAP1LC3B or MAP1LC3B2

**Table 5:** mTOR- and calmodulin-associated proteins in human MLIV brain

Protein	CB	CRB
*Ratio (MLIV/Con)*		
LAMTOR1	1.81	1.27
LAMTOR3	2.65	1.63
LAMTOR4^1^	n.d	1.73
LAMTOR5^1^	8.71	1.71
RPTOR	1.51	0.57
RICTOR	0.18	+
DEPTOR	+	1.36
MLST8	−	+
TBCK	0.37	7.35
TSC1	−	0.33
MYCBP2	n.d	0.26
CAMK2A	1.03	0.52
CAMK2B	0.38	0.53
CAMK2D	0.78	0.47
CAMK4	0.38	0.32
CAMKK1	−	0.94
CAMKK2	0.27	0.59
CAMSAP1	0.59	0.34
CAMSAP2	1.91	0.24
ITPKA	0.65	0.18
PDE1B	−	0.79

A total of 13 proteins related to mTOR signaling were detected and 23 proteins associated with calmodulin were detected. The proteins listed in the Table are those that differ between the control and MLIV. See Table 1 for explanation of the symbols

^1^ Only one peptide was identified for these proteins

Neuroinflammation in MLIV has been reported, with activation of microglia and astrocytes in *Mcoln1*^*−/−*^ mouse brain [25, 38], and we observed elevation in a number of markers such as MAC2, LGALS3BP, GFAP and TGFBI. Unexpectedly, levels of a number of proteins associated with the complement system were also elevated in the MLIV CB and CRB, and also in the CSF (Table 6), suggesting involvement of the classical complement pathway [39]. Levels of proteins related to the type 1 interferon (IFN) response (Fig. 1a, b) were also increased, with 17 of the 19 detected IFN-related proteins increased in MLIV in the CB and 14 elevated in the CRB (Table 7). These proteins include IFN-stimulated proteins such as ISG15, GBP1, IFIT1 and IFIT3. Only 5 of the IFN-related proteins were detected in the CSF, and all were similarly increased, indicating activation of the IFN response in MLIV patients.

**Table 6:** Complement system in human MLIV tissue and in *Mcoln1*^*−/−*^ mouse brain

Protein	CB	CRB	CSF	*Mcoln1*^*−/−*^ versus *Mcoln1*^±^
	Ratio (MLIV/control)			Fold-change
C1QA^1^	+	n.d	+	2.73^*^
C1QB	+	4.84	13.8	2.47^*^
C1QC	+	2.71	2.85	2.45^*^
C4A	+	+	+	2.82
C3	0.4	0.36	1.88	3.09^*^
C1QBP	1.56	1.77	1.24	1.00
CD59	1.93	1.85	1.33	1.00
CFB	−	0.15	n.d	n.d
CFH	−	0.31	−	n.d

12 proteins were detected in human brain (with 10 having > 1 peptide). See Table 1 for explanation of the symbols. Fold-change in RNA expression by RNAseq is shown for the same genes (by orthologous conversion) in *Mcoln1*^*−/−*^ versu*s Mcoln1* + ^*−/−*^ mice. ^*^, *p* < 0.05

^1^ Only one peptide was identified for this protein

**Table 7:** IFN signaling in human MLIV tissue and in *Mcoln1*^*−/−*^ mouse brain

Protein	CB	CRB	CSF	*Mcoln1*^*−/−*^* versus Mcoln1*^±^
	Ratio (MLIV/Con)			Fold Change
IRF9	+	+	n.d	3.11^*^
IFIT1	103	102	n.d	5.45^*^
IFIT2	+	2.88	n.d	2.67^*^
IFIT3	339	159	+	3.91^*^
IFIT5	1.82	1.58	n.d	n.d
IFI16	4	n.d	n.d	n.d
ISG15	+	16.62	1764	4.66^*^
MX1	+	20.57	16.1	12.1^*^
MX2	+	n.d	n.d	3.33^*^
OAS2	+	n.d	n.d	7.81^*^
GBP1	+	n.d	1.35	n.d
MNDA	+	+	n.d	4.82
STAT1	3.18	3.58	67.1	2.11^*^
STAT2	4.45	+	n.d	1.47
BST2	1.74	5.24	n.d	3.45^*^
MAVS	−	+	n.d	1.05

19 proteins were detected by MS-based proteomics in human brain (with 18 have more than 1 peptide), of which 16 are higher in MLIV. See Table 1 for explanation of the symbols. Fold-change in RNA expression by RNAseq is shown for the same genes (by orthologous conversion) in *Mcoln1*^*−/−*^* versus Mcoln1*^±^ mice. ^*^, *p* < 0.05

In addition to proteomics analysis of human brain, we also performed RNAseq on the brain of *Mcoln1*^*−/−*^ mice [10] at the end-stage of disease, i.e. at ~ 7 months of age (Fig. 1e). 184 genes were differentially expressed (DE) (fold-change ≥ 1.5, *p* adjusted ≤ 0.05) with 174 up-regulated and 10 down-regulated. When genes detected in the mouse by RNAseq overlapped with orthologous proteins detected by proteomics in the human brain, the results were remarkably similar. Thus, levels of RNA encoding complement genes were elevated in 7-month old *Mcoln1*^*−/−*^ mouse (Table 6), as were levels of IFN-related genes (Table 7), confirming the validity of the human proteomics data. The overlap between the patient and mouse datasets also highlights the strength of the *Mcoln1*^*−/−*^ mouse as an accurate pre-clinical model of MLIV. A number of additional pathways were up-regulated in the mouse RNAseq data, which were not detected in the proteomics analysis of human brain, including inflammatory genes such as cytokines and chemokines. Moreover, many of the genes and pathways that were detected in 7-month-old *Mcoln1*^*−/−*^ mice by RNAseq (or detected by proteomics analysis of human brain) were also detected at earlier pre-symptomatic stages, i.e. 1 month and early symptomatic (2/3-month old mice). Thus, even at early pre-symptomatic stages, levels of genes associated with the complement and IFN pathways were up-regulated. Likewise, microglia activation also begins early in disease development [38] as RNAseq demonstrated that *Mpeg1*, a macrophage marker expressed also in microglia, was DE in 1-month *Mcoln1*^*−/−*^ mice, as was *Cxcl10*, whose levels are also altered in models of experimental autoimmune encephalomyelitis [40].

Levels of a number of other genes, including cathepsins and genes related to sphingolipid metabolism, were also up-regulated at early stages of disease in *Mcoln1*^*−/−*^ mice, as were genes related to microglia activation [25] which included *Trem2* and *Tyrobp*, key disease-associated microglia (DAM) genes [41]. Genes associated with defective myelination were down-regulated [27] (Table 8). Defective myelination is a hallmark of MLIV pathology [25, 27] and this data indicates that changes in the expression of myelination genes, or in levels of proteins associated with myelination, are most significant at the active stage of brain myelination in early development.

**Table 8:** DEGs in pre-symptomatic *Mcoln1*^*−/−*^ mice

Gene	*Mcoln1*^*−/−*^ versus *Mcoln1*^±^ 1 month 2 −3 months		DEGs (taken from Ref [74])	DEGs (taken from Ref [53])
	*Fold-Change*			
*Cathepsins*				
*Ctss*	1.37	1.81		1.91
*Ctsc*	2.05	*1.71*		1.34
*SL metabolism*				
*Fa2h*	− 1.73	− 1.68	− 2.15	
*S1pr5*	− 1.50	− 1.61		
*Inflammation (microglia)*				
Cd68	1.78	2.47		1.68
*Lgals3bp*	*1.17*	2.37		4.56
*Mac2*	*1.16*	1.73		
*Lyz2*	1.53	2.31		2.53
*Mpeg1*	2.72	2.48		1.64
*Trem2*	1.37	2.06	1.73	1.58
*Tyrobp*	1.33	1.70	2.11	2.02
*Inflammation (chemokines)*				
*Cxcl10*	3.05	4.50		
Ccl6	*1.21*	2.09		1.62
*Complement*				
*C4b*	1.72	2.11		3.91
*C1qa*	1.32	1.67		1.63
*C1qb*	1.31	1.69		1.76
*C1qc*	*1.07*	1.66		1.56
*Interferon*				
*Irf7*	*1.02*	2.69		3.46
*Ifit1*	*1.31*	1.91	1.67	5.66
*Ifit3*	*1.35*	2.16	2.18	5.51
*Ifit3b*	*1.58*	2.58		4.65
*Ifi30*	1.43	1.63		
*Ifi27l2a*	*1.21*	1.81		4.01
*Oasl2*	*1.25*	2.49		4.89
Irgm1	*1.23*	1.83		1.83
Gbp2	*1.11*	1.95		3.05
*Myelination*				
*Mag*	− 1.80	− 1.53	− 2.12	
*Mal*	− 2.45	− 1.97		
*Mobp*	− 1.85	− 1.86		
*Mog*	− 1.96	− 1.79		
*Pllp*	− 1.67	− 1.49		
*Plp1*	− 1.72	− 1.52		
*Gjc2*	− 1.62	− 1.47		
*Gpr37*	− 1.66	− 1.61	− 1.76	
*Opalin*	− 2.28	− 1.99		
*Cnp*	− 1.86	− 1.49		
*Calcium*				
*Tmem63a*	− 1.64	− 1.65		
*Others*				
*Mcoln1*	− 8.41	− 7.83	− 1.61	− 89.26

63 genes were DE (fold-change ≥ 1.5, *p* adjusted ≤ 0.05) at 1 month of age (9 up-regulated and 54 down-regulated) and 62 genes were DE at 2–3 months of age (41 up-regulated and 21 down-regulated). Fold-changes (*Mcoln1*^*−/−*^ versus *Mcoln1*^±^) were statistically significant (*p* < 0.05) except for the values in italics. Data were compared with two published studies; cerebral cortex of 2 or 3 months old *Mcoln1*^*−/−*^ versus. *Mcoln1*^+*/*+^ mice [74] or isolated microglia from 2 month-old *Mcoln1*^*−/−*^ versus. *Mcoln1*^+*/*+^ mice ^53^. When no value is listed for the data from Refs 56 and 57, the genes were not detected in these studies

Finally, we performed MS-based proteomics on CSF from the human brain patient and from CSF obtained from the *Mcoln1*^*−/−*^ mice. In the latter, 2092 proteins were identified and quantified. 67 proteins were DE (*Mcoln1*^*−/−*^ versus *Mcoln1*^±^) at 1 month, 99 were DE at 2 months, 49 at 3 months and 162 at 7-months of age (fold-change ≥ 2, p < 0.05, number of peptides ≥ 2; not shown). While the same proteins were not detected at each of these ages, the deregulated proteins belonged to similar pathways. Thus, proteins related to a number of pathways increased, including lysosomal proteins (i.e. LAMP1, GNS, PPT1, TPP1, CTSD, CTSB, CTSL, CTSZ, PRCP), Ca^+^-binding proteins and proteins that regulate Ca^+^ levels (i.e*.* RCN1, SLC3A2, PPP3R1), proteins related to the immune system (i.e. LAG3, MAC2, LGALSL, LYZ2, RELT, TREM2 and complement C4B, C1QB, CFP), and several proteins associated with cellular trafficking (LMAN1, COPE, TOM1, VPS26A, STX7). Levels of proteins related to apolipoprotein metabolism were decreased (APOM, APOBEC2, APOA4)*.* As no common proteins were detected, we then compared all *Mcoln1*^*−/−*^mice to all *Mcoln1*^±^ mice, independent of their age. 63 proteins were DE (48 up-regulated and 15 down-regulated). Levels of the Purkinje cell protein PCP4 were increased, as were levels of most lysosomal proteins (Table 9). Levels of A1BG (alpha-1B-glycoprotein, Table 9) decreased by the largest amount in mouse CSF. A1BG levels were also decreased in the MLIV patient CSF (Table 9). A1BG has been suggested as a biomarker [42–44].

**Table 9:** Protein levels in human and mouse CSF

Protein	Human *MLIV/Con*	*Mcoln1*^*−/−*^ versus *Mcoln1*^±^
	Ratio	Fold-change
*A1bg*	0.11	− 26.0^***^
*Lysosomal proteins*		
*Epdr1*	n.d	2.62^***^
*Ppt1*	1.39	7.63^***^
*Man2b1*	n.d	3.23^*^
*Man2b2*	n.d	6.95^**^
*Gns*	+	6.72^*^
*Tpp1*	0.70	5.73^*^
*Lamp1*	n.d	2.88^*^
*Ctsz*	3.17	2.25^**^
*Calbindin-related proteins*		
*Calb1*	0.46	25.21^***^
*Calb2*	+	10.41^***^
*Pcp4*	+	12.7^***^

Protein levels were measured in the CSF from the MLIV patient and from 1-, 2-, 3- and 7-month-old *Mcoln1*^*−/−*^ versus *Mcoln1*^±^ mice (n = 3–4 for each time point). Changes are indicated by + for a protein that was detected only in the MLIV sample and by n.d. when it could not be detected in either sample.* *p* < 0.05, ** *p* < 0.01, *** *p* < 0.001

Three of the top 5 up-regulated proteins in the CSF from *Mcoln1*^*−/−*^ mice are related, namely calbindin 1 (CALB1), calbindin 2 (CALB2) and PCP4 (Table 9). Strikingly, levels of CALB2 and PCP4 were elevated in the CSF from the human MLIV patient; CALB2 and PCP4 were not detected in the CSF of the control human brain. Calbindins have been suggested to act as biomarkers in diseases with cerebellar involvement [45] including LSDs [46, 47]. To determine whether either of these proteins might be a suitable biomarker in human MLIV, we analyzed levels of CALB2 in CSF; note that due to the limited amount of CSF available to us, we were only able to evaluate levels of one of these two proteins. By ELISA, CALB2 levels were 2247.5 pg/ml CSF in the control patient and 2838.5 pg/ml in the CSF from the MLIV patient. Likewise, levels of CALB2 were elevated in mouse CSF as early as 1 month of age, and detected in 69% of *Mcoln1*^*−/−*^ mice but in only 12% of *Mcoln1*^±^ mice (Table 10), suggesting that CALB2 might indeed act as a biomarker for MLIV which could also be used to test the efficacy of therapies as and when they become available.

**Table 10:** CALB2 levels in mouse CSF

Age of mice	*Mcoln1*^±^		*Mcoln1*^*−/−*^	
Month	Not detected	Detected	Not detected	Detected
1	4	0	1	2
2	4	0	1	3
3	2	2	1	2
7	4	0	1	2
*Total*	14	2	4	9

Protein levels detected by MS-based proteomics were measured in the CSF from 1-, 2-, 3- and 7-month-old *Mcoln1*^*−/−*^ versus *Mcoln1*^±^ mice. The numbers represent the number of mice in which CALB2 was detected in the CSF

## Discussion

Until the current study, the vast majority of information on MLIV neuropathology was obtained from clinical observation [48], studies in cell culture [36] or from study of the *Mcoln1*^*−/−*^ mouse [10]. The current study extends this data by analyzing the first available human brain sample from an MLIV patient. Having said that, our study is somewhat unconventional inasmuch as we make conclusions about pathophysiological pathways in MLIV based on a proteomics study comparing only one human brain sample with one control, although proteomics was performed on two different brain areas, namely the cortex and cerebellum, and on the CSF. Since this is the first time that such tissue has been made available for research purposes, and despite the obvious limitations of this approach, we are of the opinion that such a study is warranted and of value for both the research, clinical and patient communities associated with MLIV. Nevertheless, conclusions must be drawn with suitable caution taking into account the minimal sample size.

**Fig. 2:**
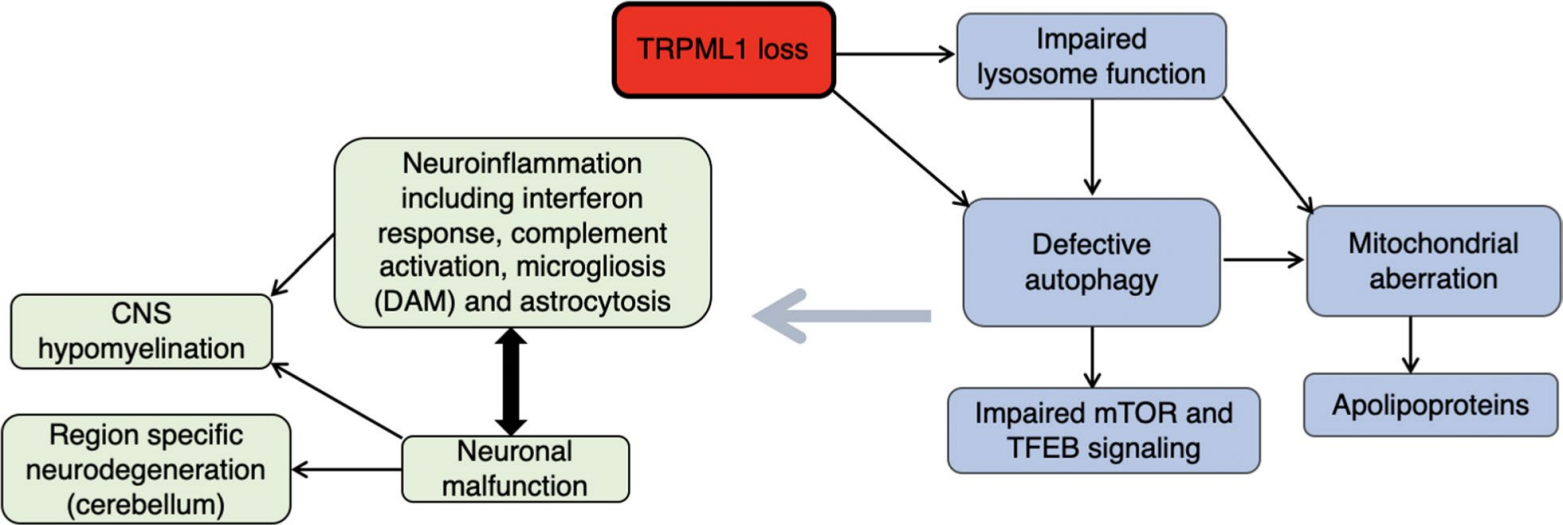
Pathological pathways in MLIV. Loss of the lysosomal channel TRPML1 leads to impaired lysosome function, which in turn affects autophagy, mitochondria including apolipoproteins and mTOR signaling. MLIV patients suffer from neuroinflammation, neuronal malfunction and hypomyelination, all eventually leads to neurodegeneration late in the disease course. For further details, see text

A broad overview of the pathophysiology in MLIV suggests that impairment of lysosome-related cellular pathways leads to brain pathology and changes in neurological function (Fig. 2). Since TRMPL1 encodes for a late endosomal/lysosomal channel involved in regulation of a broad array of lysosomal processes, the effect of reducing its activity might be more general than that seen in LSDs which are caused, for instance, by the loss of a specific lysosomal hydrolase. Thus, loss of TRPML1 leads to more wide-ranging changes in lysosomal function (supported by changes in levels of a large number of lysosomal proteins in the current study, along with defective autophagy (Fig. 2)). Since the brain tissue was taken post-mortem, it is obviously not possible to define which pathways are primary or secondary, but since TRMPL1 directly impinges upon the lysosome, it is reasonable to assume that the lysosome is first affected. Inhibition of autophagy in MLIV is likely a result of inhibited lysosomal function, and in turn may lead to dysregulation of upstream mTOR and TFEB signaling (Fig. 2), although recent studies [49] on the role of TRPML1 in regulation of TFEB suggest that changes in TFEB signaling could also be a direct result of TRPML1 loss. The temporal relationship between these changes and mitochondrial abnormalities, along with changes in lipoprotein metabolism, cannot be directly established in the current study, but the relationships between autophagy and mitochondrial defects are well documented [50, 51]. These altered cellular pathways lead to a complex course of neuropathophysiological changes, including aberrant neuronal function and neuroinflammation, with the latter including the interferon response, complement activation, microgliosis and astrocytosis (Fig. 2). The time-course of these events cannot be determined in autopsy MLIV samples, although a number of these pathways are altered at the early stages in disease development, at least in the mouse model, as ascertained by the RNAseq data, and are therefore likely to be the direct consequence of the loss of TRPML1.

While a number of these pathways (Fig. 2) were previously implicated in MLIV pathophysiology, we now provide a more comprehensive picture of some of them and identify a number of new proteins that were not previously known to be identified with MLIV. For instance, levels of a number of lysosomal hydrolases were increased. Earlier studies had shown similar findings, including studies in fibroblasts derived from MLIV patients [23, 52]. The elevation we report in cathepsin levels is similar to that observed in *Mcoln1*^*−/−*^ mice microglia [53], in TRPML1-deficient cells [54] and in astrocytic cultures [38], as is the elevation in LAMP1 [9, 23, 38]. Thus, the increase in levels of lysosomal proteins is consistent with the enlarged lysosomes reported in MLIV retina [55].

The changes in apolipoproteins are much more extensive than previously reported, since only changes in ApoE were reported in MLIV microglia [53] and in astrocytes from *Mcoln1*^*−/−*^ mice [38]. We now report a significant increase in levels of APOD in the CB and CRB. APOD is mainly expressed in glia and neurons [56] and is elevated in some other neurological disorders such as Alzheimer’s disease, specifically in the CSF [57], in multiple sclerosis [58], in the aging brain [59] and in neuronal tissues from Niemann Pick C disease [60]. APOD and APOE have also been implicated in Parkinson’s disease [61]. APOD and APOE, are involved in the removal of lipids during nerve cell degeneration [62], which may be of importance in demyelination. Moreover, levels of two PLIN proteins (PLIN 3 and 4) were elevated in the MLIV brain and in neuronal forms of Gaucher disease [63, 64]. Lipid droplets have been implicated in neurodegeneration [65, 66] including in Parkinson’s disease, which suggest that they play a broad role in neurodegenerative diseases.

Likewise, we observed down-regulation of proteins associated with trafficking between the ER, Golgi and the lysosome, in line with the defects in retrograde trafficking of late endosome and lysosome to Golgi retrograde trafficking that were reported in TRPML1-deficient cells [67, 68].

Defective intracellular trafficking can be reflected in changes in autophagic pathways. Previous studies in neuronal cultures from *Mcoln1*^*−/−*^ mice demonstrated increased levels of the classic autophagic marker, LC3-II [69], but our current data allows us to further dissect autophagy pathway inasmuch as a number of the autophagosome markers, i.e. ATG proteins, which control autophagosome formation [32], are decreased. While interpretation of the LC3 readout in the post-mortem tissue is limited due to inability to track the kinetics of the autophagy process, increased levels of LC3 in the context of inhibited lysosomal function and decreased expression of the proteins involved in the upstream stages of autophagosome formation, clearly indicate block of autophagy [70]. Increased levels of LC3 in this case result from diminished degradation of autophagosomes on lysosomes due to inhibited lysosomal function. TRPML1 is known to induce autophagosome biogenesis through a signaling pathway involving activation of calcium/calmodulin-dependent protein kinase β [49], consistent with our observation of decreased levels of CAMKK proteins in the MLIV patient brain. Likewise, TRPML1 overexpression leads to mTOR inhibition and activation of its targets, including TFEB and ULK1 [36]; however, we cannot validate changes in TFEB activation in our study since it was not detected by the proteomics analysis.

Neuroinflammation, activation of the complement system, and defective myelination were also observed in the MLIV brain. For the former, noticeable pathways of neuroinflammation include activation of the interferon pathway, as previously demonstrated in *Mcoln1*^*−/−*^ microglia [53] and astrocytes [38], and similar to that previously observed in another LSD, Gaucher disease [63, 71]. However, in Gaucher disease, the interferon pathway was categorized as a secondary disease pathway, since eliminating this pathway had no effect on the course of the disease in mice [56]; whether this is also the case in MLIV is currently unknown.

Since we were also able to obtain small amounts of CSF from the human MLIV patient, we performed a preliminary analysis to determine whether changes in the brain might be reflected in the CSF, and thus indicate that analysis of CSF might be useful to identify biomarkers. The CSF proteomics data was largely consistent with the brain data, but less so with CSF aspirated from *Mcoln1*^*−/−*^ mice. However, one specific protein detected in both human and mouse CSF, namely CALB2, was significantly elevated in both the proteomics analysis and was confirmed by ELISA, similar to that observed in late infantile neuronal ceroid lipofuscinosis type 2 (CLN2) and CLN3 patients [47]. Due to the low amount of CSF available to us, we were unable to verify whether CALB1 might also act as a biomarker, but CALB1 has also been suggested as a biomarker in Niemann-Pick type C1 [46]. Thus, while not yet being fully validated, our data suggest that further analysis of CALB1 and CALB2 might be worthwhile in light of their potential as a biomarker for MLIV.

## Conclusions

We analyzed the proteome in two brain regions from an MLIV patient and a healthy aged- and gender-matched control, and annotated the observed changes in protein levels with changes previously observed in in vitro and in vivo models of MLIV*.* Our data confirm the usefulness of the *Mcoln1*^*−/−*^ mouse as a genuine model of MLIV, and more importantly, shed light on some new putative players in MLIV pathology, such as apolipoproteins, lipid droplets, the IFN pathway, disease-associated microglia activation and autophagy. Finally, analysis of CSF from both the human patient and from *Mcoln1*^*−/−*^ mice suggest that CALB1 and CALB2 might be useful as biomarkers to help chart the course of disease development and the outcomes of pre-clinical and clinical trials.

## Data Availability

The RNAseq dataset generated during the current study was deposited in the Gene Expression Omnibus (GEO) database, www.ncbi.nlm.nih.gov/geo (accession no. GSE157825). Mass spectrometry data was deposited to the ProteomeXchange Consortium via the PRIDE [72] partner repository with the dataset identifiers PXD020490, PXD020491 and PXD020494.

## Abbreviations

Calb: Calbindin
cAMP: Cyclic adenosine monophosphate
CB: Cerebellum
CNS: Central nervous system
CRB: Cerebral cortex
CSF: Cerebrospinal fluid
DAM: Disease-associated microglia
DE: Differentially-expressed
DEGs: Differentially-expressed genes
IFN: Interferon
LSD: Lysosomal storage disease
MS: Mass spectrometry
mTOR: Mechanistic target of rapamycin
MLIV: Mucolipidosis type IV
SL: Sphingolipid
TFEB: Transcription factor EB
TPC: Two pore channels
TRPML1: Transient receptor potential channel
